# A Description–Experience Framework of the Psychology of Risk

**DOI:** 10.1177/17456916211026896

**Published:** 2021-12-07

**Authors:** Ralph Hertwig, Dirk U. Wulff

**Affiliations:** 1 Center for Adaptive Rationality, Max Planck Institute for Human Development, Berlin, Germany; 2 Center for Cognitive and Decision Sciences, University of Basel

**Keywords:** description–experience gap, information sampling, probability weighting, risk behavior, risk communication, risk perception

## Abstract

The modern world holds countless risks for humanity, both large-scale and intimately personal—from cyberwarfare, pandemics, and climate change to sexually transmitted diseases and drug use and abuse. Many risks have prompted institutional, regulatory, and technological countermeasures, the success of which depends to some extent on how individuals learn about the risks in question. We distinguish between two powerful but imperfect teachers of risk. First, people may learn by consulting symbolic and descriptive material, such as warnings, statistics, and images. More often than not, however, a risk’s fluidity defies precise description. Second, people may learn about risks through personal experience. Responses to risk can differ systematically depending on whether people learn through one mode, both, or neither. One reason for these differences—and by no means the only reason—is the discrepancy in the cognitive impact that rare events (typically the risk event) and common events (typically the nonoccurrence of the risk event) have on the decision maker. We propose a description–experience framework that highlights not only the impact of each mode of learning but also the effects of their interplay on individuals’ and collectives’ responses to risk. We outline numerous research questions and themes suggested by this framework.

Some stories have to be experienced to fully grasp—the Korea crisis is one of them. I arrived in Seoul on the evening of May 28. As I was dressing for breakfast the next morning, I was jarred by a news alert ringing on my phone: North Korea had just fired a short-range ballistic missile that had landed in the sea off its east coast.

I waited for the sirens to tell us to go to the hotel shelter, as happened when I was in Israel during a Hamas rocket attack. But there were no sirens. Nothing. The breakfast buffet was packed. The mood was: Another North Korean missile test? Oh, pay no attention to our crazy cousins. *Could you pass the kimchi, please?*

—Friedman (2017; paras. 1 and 2)

As Friedman’s (2017) eyewitness testimony illustrates, people’s understanding of and behaviors toward risks can sometimes be perplexing. One key factor in South Koreans’ nonchalance about missile tests may be their experience of living in the shadow of North Korea’s nuclear threat: Between the first test in 1984 and March 2020, North Korea carried out 147 missile tests, conducted six nuclear test explosions, and repeatedly verbally abused its neighbor to the south (Arms Control Association, 2020). Having experienced more than 3 decades of tests and bluster, most South Koreans seem to agree that a barking dog never really bites. Yet despite collectively shrugging at missile launches, South Koreans responded vigilantly and forcefully to the COVID-19 threat while most other countries and their citizens were still idling (Sang-Hun, 2020).^1^ According to *The Wall Street Journal*, the “key to South Korea’s success came from blending technology and testing like no other country, centralized control and communication—and a constant fear of failure” (T. W. Martin & Yoon, 2020). Many observers pointed out that these rapid and concerted efforts were probably a result of the country previously having found itself on the brink of a pandemic after an outbreak of the Middle East respiratory syndrome coronavirus (MERS-CoV) in 2015, when the authorities’ blundering response—characterized by government secrecy, poor infection control, and a general lack of preparedness—sparked panic (Jack, 2015).

Experience matters. Yet it is not the only way of learning about risks and their magnitude. Another powerful teacher is description in the form of written warnings, media coverage, health and accident statistics, fact boxes, or infographics. Figure 1 shows a paradigmatic description-based form of risk communication, a fact box. Fact boxes are simple tools that summarize the best available evidence about the harms and benefits of a medical intervention or health behavior—here, the prescription of antibiotic treatment in children with an acute middle-ear infection (Brick et al., 2020)—in table form. Like experience-based learning, learning about risks from descriptions can also trigger a variety of responses, from compliance to backfire effects (Andrews, 2011; Steinhart et al., 2013).

**Fig. 1.**
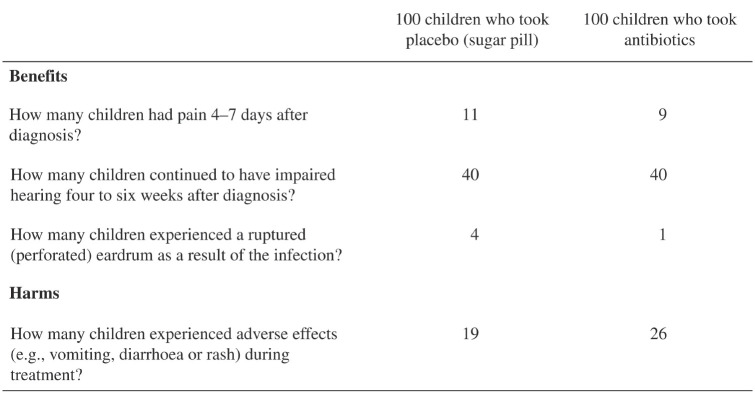
Example of a fact box. A fact box is a simple tabular summary of the best available evidence about the benefits and harms of a medical procedure, treatment, or health behavior. This fact box describes the outcomes for children ages 0 through 15 years with an acute middle-ear infection who received either antibiotics or placebo (sugar pill) for 7 to 14 days. Adapted from Brick et al. (2020). Original figure published under a Creative Commons Attribution License (CC-BY-4.0).

As these examples show, the human response to risk is not of one kind. People respond in distinct and sometimes bewildering ways to one and the same risk; their responses can change over time, and behaviors can comply with or fly in the face of expert recommendations. We propose that one insight that can help discern a structure behind this behavioral diversity is that laypeople and experts alike learn about risks and their magnitude via two distinct modes: description and experience. In some circumstances, people have recourse to both learning modes; in others, they must rely on just one. For instance, physicians can both consult health statistics to evaluate the risks of a medical intervention and draw on their own experience of treating patients and monitoring the effects of the intervention in question. Patients, on the other hand, initially have no experience with the intervention; they can refer only to summary descriptions of benefits and harms such as fact boxes (Fig. 1); with time, however, they move from being an “experiential blank slate” to an “experiential authority” (e.g., experiencing the consequences of long-term use of strong opioids; Schulte et al., 2021). Furthermore, the lessons that description and experience convey do not necessarily converge; they sometimes contradict each other. The opinions of experts and laypeople often diverge when one group draws on experience and the other on descriptions of risks. We suggest that research into the description–experience distinction and the effects of these two powerful but imperfect teachers of risk will help provide a clearer picture of how people perceive and respond to risk.

This description–experience view on human responses to risk complements other frameworks of risk perception, such as the psychometric approach (Slovic, 1987), social amplification of risk (Kasperson et al., 1988), and information framing (Peters et al., 2011), by drawing attention to the process of learning about risks and to the statistical properties of risk events (see also Rogers, 1997). To shed light on learning and the sometimes collaborative, sometimes competitive interplay of symbolic descriptions and personal experience, we turn to a long-standing line of research: risky choice between monetary gambles. Although our ultimate concern is with the psychological response to real-world risks, and not with choice in experimental contexts, this line of research has accumulated valuable evidence for predicting human response to risk, particularly through its innovative investigation of the description–experience gap.

## The Description–Experience Perspective in Risky Choice

There is a time-honored tradition of using monetary gambles to examine how people respond to risk—whether by means of thought experiments (e.g., Bernoulli, 1738/1954) or behavioral experiments (e.g., Allais, 1953). This fondness for gambles is understandable. Monetary gambles embody what many consider to be the building blocks of real-world choice options: an option’s potential outcomes and the probabilities of those outcomes (Lopes, 1983). These are the pillars of influential choice models such as expected value theory, expected utility theory, and cumulative prospect theory (CPT). When studying human choice between monetary gambles, most scholars have relied on gambles in which all information about the options’ outcomes and their probabilities is explicitly stated or symbolically represented (e.g., in pie charts). Consider the following gamble pair (from Kahneman & Tversky, 1979):

A: 50% chance to win 1,000 [shekels], 50% chance to win nothing

B: 450 [shekels] for sure

All possible information is stated, leaving people to make a *decision from description* (see Hertwig & Erev, 2009). Such decisions from description are sometimes also possible in the real world: Weather forecasts, actuarial tables, and mutual-fund brochures all offer descriptions of possible outcomes and their probabilities. Yet many human behaviors—falling in love, interviewing for jobs, crossing the street—come without a manual detailing the possible outcomes and their probabilities. Instead, people can draw on their personal experiences, thereby making *decisions from experience* (Hertwig, 2015; Hertwig & Erev, 2009; Wulff et al., 2018).

### The description–experience gap

**Fig. 2.**
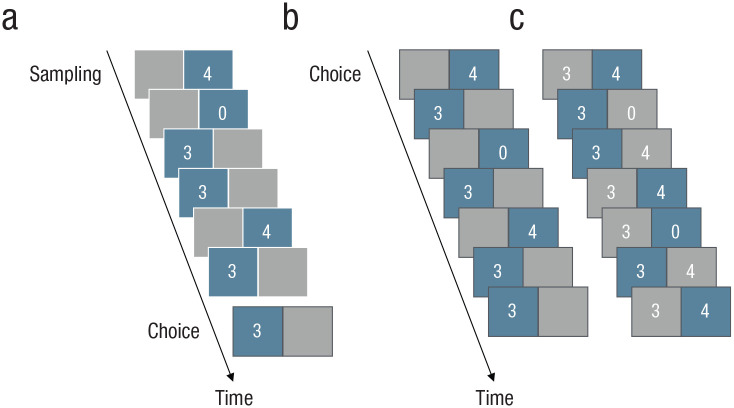
Three main paradigms for investigating decisions from experience. The sampling paradigm (a) consists of an initial sampling stage (represented by six fictitious draws, shown in blue) in which a person explores two payoff distributions without costs by clicking on one of the two buttons on the computer screen. After completing sampling, the person sees a choice screen (framed in black) and is asked to make a final choice. The partial-feedback paradigm (b) collapses sampling and choice; thus, each draw represents an act of both information-seeking (exploration) and taking advantage of what one has learned (exploitation). The full-feedback paradigm (c) is identical to the partial-feedback paradigm except that it also provides feedback on the forgone payoff—that is, the payoff the person would have received had they chosen the other option (shown in gray). Copyright © 2018 by the American Psychological Association. Adapted with permission from “A Meta-Analytic Review of Two Modes of Learning and the Description–Experience Gap,” by D. U. Wulff, M. Mergenthaler-Canseco, and R. Hertwig, 2018, *Psychological Bulletin*, *28*(2), Figure 1, with permission from the American Psychological Association.

Experimental research on decisions from experience has typically involved a simple experimental tool: a “computerized money machine” (Wulff et al., 2019; Wulff & Hertwig, 2019) in which people are usually shown two buttons on a computer screen, each one representing an initially unknown payoff distribution. Each click of a button implements a random draw from one distribution (such as option A or B in the choice problem above). Figure 2 shows the three main variants of this money machine (see Hertwig & Erev, 2009). The choices that people make in these experimental paradigms are then compared with choices made from stated outcomes and probabilities (decisions from description).

**Fig. 3.**
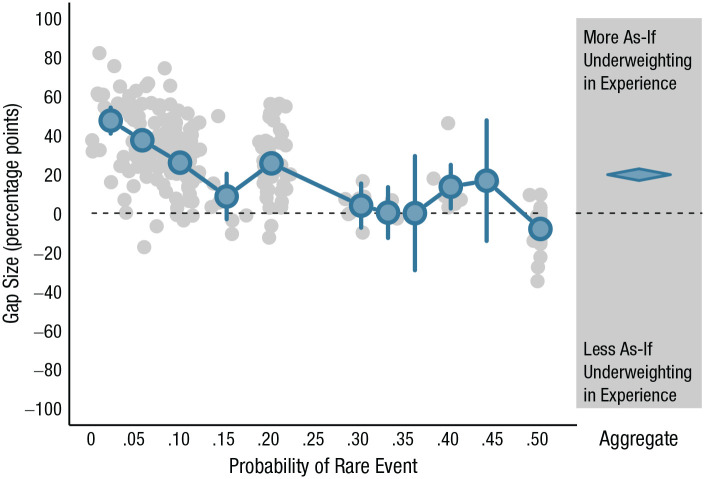
The description–experience gap as a function of the probability of a rare event. The graph displays differences between the percentage of experienced-based choices and percentage of description-based choices (assuming discrete underweighting of rare events; for details, see Wulff et al., 2018). Positive gap sizes indicate more as-if underweighting in experience than in description. The blue dots show the aggregate results in bins of size .04 defined by the true probability of the rare event. If both options contained rare events, only the rarer of the two was considered. Error bars represent 95% confidence intervals. The diamond represents an aggregate estimate and standard error as calculated from a random-effects meta-analysis.

As a substantial body of research has shown, decisions from description and decisions from experience can lead to systematically different choices. Although there are different ways to define and illustrate this description–experience gap (see Rakow & Newell, 2010; Wulff et al., 2018), the results are surprisingly similar. One approach is shown in Figure 3, which plots the differences in percentages of choices from experienced-based and description-based gambles (assuming an operationalization of the gap in terms of discrete underweighting; see Fig. 3 in Wulff et al., 2018) across a large set of choices with different outcomes and probabilities (in the sampling paradigm). Choice behavior in experience and description differ systematically as a function of the true probability of the rare outcome in the choice problem: In decisions from description, people choose as if they give *more* weight to (“overweight”) rare events than these events deserve in light of their objective probabilities. For illustration, consider a choice between two options:

A: 10% chance to lose 32, 90% chance to lose nothing.

B: 100% chance to lose 3.

In this choice, most people choose B, the safe option (e.g., Hertwig et al., 2004), consistent with the weighting of rare events as postulated by CPT (Tversky & Kahneman, 1992). Here, overweighting the 10% chance of losing 32 renders option A less attractive than it would be otherwise, thus boosting the relative attractiveness of option B. Conversely, in decisions from experience, people choose as if they give *less* weight to (“underweight”) rare events than they deserve in light of their objective probability. In the choice task above, this translates into most people choosing the risky option A (Hertwig et al., 2004) and thus acting as if they underweight the chance of losing 32. Let us emphasize that the notion of weighting as used in this illustration is meant in an as-if sense (i.e., people choose *as if* rare events had more or less impact than they deserve). Furthermore, as-if weights refer to the objective probabilities of the outcome distributions and not to the relative frequencies with which people actually experience the events (see also Regenwetter & Robinson, 2017); in sampled experience, objective probabilities and experienced frequencies can differ. Having said this, the as-if weighting pattern observed in decisions from experience has attracted much attention because it contradicts the pattern assumed in CPT. CPT’s key assumption of overweighting of low-probability events has been invoked to explain a wide range of tangible real-world behaviors, such as the purchase of lottery tickets (suggesting overweighting of the low probability of a *win*) and—somewhat paradoxically because the two behaviors signal opposite risk attitudes—the purchase of insurance policies (suggesting overweighting of the low probability of a *loss*; e.g., Camerer, 2000).

The systematic gap in choice percentages from experienced-based and description-based gambles can be substantial (see Fig. 3); for probabilities of 5% and lower, the average gap amounted to 13.3 percentage points. More generally, Wulff et al.’s (2018) meta-analysis of studies with the sampling paradigm found that when a choice involves a risky and a safe option—the type of choice often used to measure risk preference behaviorally in economics and psychology (see Hertwig et al., 2019)—the average gap size amounts to 18.7 percentage points; when a choice involves two risky options, the gap is 7 percentage points. Similar gap sizes of 22.7 and 7.9 percentage points, respectively, for the two problem types have been observed for the partial-feedback paradigm (see Wulff et al., 2018, Appendix C). Furthermore, the description–experience gap is not limited to risky choice. It has also been observed in diverse reasoning and choice domains, including intertemporal choice (Dai et al., 2019), social interaction in strategic games (Isler et al., 2020; J. M. Martin et al., 2014), ambiguity aversion (Dutt et al., 2014; Güney & Newell, 2015), consumer choice (Wulff et al., 2015), financial risk-taking (Lejarraga, Woike, & Hertwig, 2016), medical judgments and decisions (Armstrong & Spaniol, 2017; Fraenkel et al., 2016; Lejarraga, Pachur, et al., 2016; Wegier & Shaffer, 2017), adolescent risk-taking (van den Bos & Hertwig, 2017), categorization (Nelson et al., 2010), confidence estimates (Camilleri & Newell, 2019; Lejarraga & Lejarraga, 2020), causal reasoning (Rehder & Waldmann, 2017), and visual search (Zhang & Houpt, 2020).

These findings suggest that the distinction between description and experience is relevant for cognition and behavior more generally, although not all of the above instances are necessarily characterized by overweighting and underweighting of rare events (see, e.g., Lejarraga, Woike, & Hertwig, 2016; van den Bos & Hertwig, 2017). The systematic gap in the implied impact of described and experienced rare events can thus be expected to be relevant for risk perception and behavior beyond monetary gambles to the extent that rarity is a key property of real-world risks (see also J. M. Martin et al., 2014). Pandemics, serious side effects of vaccination, and car accidents are—fortunately—all rare events, although their fatality rates may follow different distributions (e.g., thin-tailed vs. fat-tailed; Cirillo & Taleb, 2020) and affect individuals or collectives. Before we explore the implications of the description–experience distinction for risk perception and risk behavior, let us take a closer look at some key properties of description and experience.

### Attributes and ambiguities of description and experience

In this section, we draw on Hertwig et al.’s (2018) conceptual (and admittedly incomplete) characterization of description and experience as two major paths to knowledge. In their view, descriptions can be understood as externalized symbolic representations of knowledge—written or spoken words, numbers, or images that can pertain to any kind of knowledge (e.g., propositional, causal, procedural, or episodic). Of particular relevance in the context of risk communication, descriptions need not be bounded by time and place and can inform individuals about unlikely events, things that have not (yet) materialized (e.g., the long-term consequences of climate change), or things that nobody will ever experience (Pinker, 2007). People can also refer to descriptions to learn about the possible detrimental consequences of actions without needing to pay the price of experiencing them. Learning from description is one of the key engines of cultural evolution and the sharing of knowledge (Richerson & Boyd, 2005) and is a quintessentially human competence.

Descriptions are abstractions in that they necessarily reduce and summarize the multidimensionality of individual or collective experience; without such reduction they could not be efficient representations of knowledge. But not all abstractions of the world are descriptions: Internal representations in terms of mental models, for instance, are not descriptions. Yet when those representations travel from the mind of the individual to the world in the form of stories, warnings, or testimony, they become descriptions and thereby accessible to others. Descriptions need authors—speakers, writers, or users of symbols. Whereas the spoken word fades quickly, the written word may be permanent. Moreover, the mere symbolic presentation of an event can (unduly) increase its psychological impact—a phenomenon again of particular relevance in the context of risk communication that Erev et al. (2008) referred to as the *mere-presentation effect*. For instance, Gregory et al. (1982) demonstrated that asking people, through the use of external representations, to imagine certain events (e.g., committing an armed robbery and getting arrested) led to an increase in the estimated likelihood of those events and sometimes even influenced people’s behavior. Relatedly, Hasher et al. (1977) observed that confidence in the truth of an assertion increases after the repeated presentation of that assertion, independent of its truth or falsity (Hertwig et al., 1997, dubbed this the *reiteration effect*). In short, despite their key role in the transfer of knowledge, descriptions are not without ambiguities. They typically require interpretation and can sometimes be misleading. Gigerenzer et al. (2007) highlighted some paradigmatic cases of misleading descriptions in the communication of health risks.

Experience is the process and result of living through events (see Hertwig et al., 2018; March, 2010). It can have physiological (e.g., pain or pleasure), cognitive (e.g., information), and subjective (e.g., unpleasantness) aspects; sometimes it has predominantly informational value and sometimes informational and material effects co-occur and conflict (i.e., the exploration–exploitation trade-off; Sutton & Barto, 1998; see also Fig. 2). Experience can be used to evaluate past actions and guide future ones (March, 2010). Positive experience with an option increases the probability of that option being chosen in the future; negative experience has the opposite effect (Denrell & March, 2001). Although undergoing an experience may require effort, learning from experience is often relatively effortless and immediately authoritative for the individual concerned. Organisms automatically make inferences, abstractions, or generalizations on the basis of their experiences. Sometimes gathering experience of the risk of harm is voluntary (e.g., going downhill skiing and potentially getting injured); sometimes the environment imposes the risk (e.g., experiencing the health risks of record temperatures during a heat wave).

Although anchored in the reality of the individual, the interpretation of experience can be ambiguous for several reasons (March, 2010), such as noise resulting from errors in observation, truly stochastic structures in the world, and the importance of learning not only from actual events but also from events that could have occurred but did not. Among the many open issues surrounding experience is the question of what should count as personal experience and, relatedly, to what extent vicarious experience has the attributes of personal experience. A vicarious experience is commonly understood as an empathetic state in response to the observation of others’ sensations, emotions, and actions (Keysers & Gazzola, 2009). Vicarious experiences appear to recruit neural processes similar to those involved in the primary experience of a sensation, emotion, or action (e.g., Singer et al., 2004).

Another source of ambiguity is that experience typically represents a momentary sample. Just how representative this sample is for the risk event in question depends on many factors, including the statistical structure of the risk event (e.g., Cirillo & Taleb, 2020), people’s ability to take into account biases in the sampling process and the sample (e.g., Fiedler & Juslin, 2006; Hogarth et al., 2015), the temporal dynamic of the risk event (e.g., immediate or delayed consequences or gradual change in the risk), the extent to which people are disinterested observers or act in the pursuit of goals that may affect a risk’s likelihood (Le Mens & Denrell, 2011), and the strength and detectability of an experiential signal (e.g., rising yearly temperature) relative to the noise of random fluctuation around a central trend (Weber & Stern, 2011).

Let us conclude by emphasizing that although we have presented description and experience in terms of a dichotomy—one that carries substantial heuristic value—we actually see them as poles on a continuum. For example, learning on the basis of a description can also form an experiential episode. It follows that some descriptive formats retain more of the qualities of the original experience than others (e.g., stories vs. statistics; natural frequencies vs. probabilities; consumer reviews vs. ratings; see also Wulff et al., 2015).

## The Description–Experience Distinction and a Fourfold Pattern of Epistemic States

In principle—if we work with the premise of a description–experience dichotomy (see Hertwig et al., 2018)—there are four epistemic states in which people can find themselves when faced with a decision involving risk. In this section, we outline these epistemic states and draw on empirical findings on the description–experience gap to suggest specific regularities in people’s responses to risk within each of those states. This exercise in extrapolation promises to yield interesting insights and to reveal research questions that would benefit from systematic investigation.

**Fig. 4.**
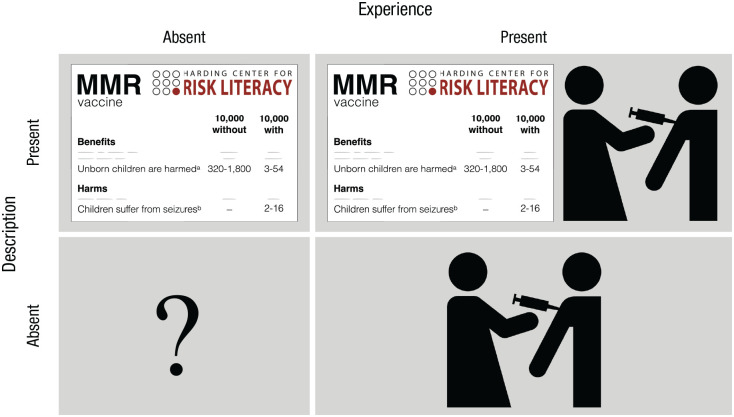
Fourfold pattern of epistemic states for the risk event of vaccinating one’s child against measles, mumps, and rubella (MMR). The fourfold pattern arises from the absence/presence of experience (vaccination icon) or description (fact box) of the benefits and harms of vaccination. The stylized MMR fact box was modeled on the original MMR fact box (Harding Center for Risk Literacy, 2016) with permission from the Harding Center for Risk Literacy.

Consider the decision of whether to vaccinate a child against measles, mumps, and rubella (MMR). Both options—to vaccinate or not to vaccinate—carry potential benefits and harms. Parents and physicians can learn about the statistical probabilities of these outcomes through description, experience, both, or neither. The result is a fourfold pattern of epistemic states (see Fig. 4).

### Description only: as-if overweighting of rare events

The first epistemic state (Fig. 4, top left) could represent the knowledge state of a first-time parent who lacks personal experience with the probabilistic consequences of having a child vaccinated and therefore needs to consult descriptions of possible outcomes and their probabilities. But not all descriptions are the same. Let us assume that a parent is unwittingly directed—via algorithm recommender systems or through motivated reasoning—to vaccine-critical websites that focus on severe reactions to vaccination (see Betsch et al., 2010), including the refuted link with autism (Taylor et al., 2014). Generalizing the as-if probability-weighting pattern introduced earlier, rare risks will loom larger than they should in light of their objective probabilities. For instance, two to 16 children in every 10,000 receiving the MMR vaccination (*p* = .0002–.0016) are reported to experience febrile seizures (Harding Center for Risk Literacy, 2016). All other things being equal, parents exposed to this information may overweight the rare harm of the vaccine relative to its linear weighting and be more inclined to decide against vaccinating their child. Alternatively, parents may come across a fact box (as introduced in Fig. 1) that may also report other relatively low-probability risks, such as how many unborn children are harmed by rubella between the 12th and 18th weeks of pregnancy. It is estimated that this happens in 320 to 1,800 unborn children out of every 10,000 people *without* the MMR vaccination who are exposed to the rubella virus, relative to three to 54 children for those *with* the vaccination. Assuming that the relatively rare risk of an unborn child harmed by the rubella virus (without the parent being vaccinated) and the rare risk of harm triggered by the vaccination are now both overweighted (all other things being equal), the psychological impact of the latter will no longer be selectively amplified.

From a public-health point of view, the overweighting of rare side effects in the vaccination scenario is an undesirable outcome. However, overweighting rare events can also result in desirable policy outcomes. Consider the risk of secondhand smoke, which is estimated to have caused more than 7,300 lung-cancer deaths in the United States each year from 2005 to 2009 (Centers for Disease Control and Prevention, 2018). This is a relatively rare outcome given that approximately 58 million people in the United States were exposed to secondhand smoke between 2013 and 2014 (Tsai et al., 2018). Explicit descriptions of the threat of secondhand smoke and its risks (e.g., lung cancer) may lead people to overweight these relatively rare risks. This, in turn, may make smokers, nonsmokers, and policymakers more likely to act—for instance, by endorsing restrictions on smoking areas.

Not all descriptions contain information on probabilities. Consider a simple warning that secondhand smoke is detrimental to a person’s health. According to support theory (Rottenstreich & Tversky, 1997), the *judged* probability (or frequency) of health risks of secondhand smoke will, all other things being equal, increase when this generic warning is unpacked into its components (sudden infant death syndrome, asthma attacks, lung cancer, heart disease, and so forth; Centers for Disease Control and Prevention, 2019b). People would thus tend to overestimate the likelihood of each component risk relative to the probability of the inclusive event “health risks of secondhand smoke.” In principle, as-if overweighting of stated small probabilities and overestimating of small probabilities of stated events (e.g., lung cancer due to secondhand smoke) can collude to boost the psychological impact of a rare risk (see also Slovic, 2000a, 2000b; Viscusi, 1990; Viscusi & Hakes, 2008). When this amplified risk represents a harm, all other things being equal, people will be more risk-averse than they would be otherwise.

### Experience only: varying weighting patterns of rare events

**Fig. 5.**
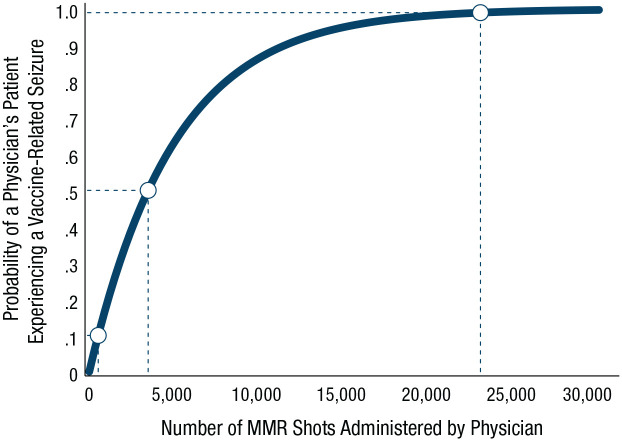
What rarity means in experience. The graph shows the probability that a physician would experience a child having a side effect of a vaccine-related seizure as a function the number of measles, mumps, and rubella (MMR) vaccine shots administered (assuming a prevalence of two in 100,000; Harding Center for Risk Literacy, 2016).

The second epistemic state (Fig. 4, bottom right) could represent the knowledge state of physicians who prefer to ignore statistics in favor of making decisions on the basis of their own experience of administering the MMR vaccine. How much experience is required for such physicians to encounter the side effect of a vaccine-related seizure a single time? Figure 5 plots the data. On average, physicians would need to administer 3,466 MMR shots (assuming a prevalence of two in 10,000; Harding Center for Risk Literacy, 2016) to experience a child having a vaccine-related seizure with 50% probability. To experience this side effect with near certainty (99%), they would have to administer a whopping 23,024 MMR shots. Because the side effects of the MMR vaccine are very rare, the physician is thus unlikely to have ever experienced them firsthand.

#### As-if underweighting of rare events

Generalizing the common probability-weighting pattern from research on the description–experience gap to this epistemic state, one may expect this physician to behave as if they underweight rare risks (and possibly delayed risks; De La Maza et al., 2019). Their experience tells them that rare events are indeed rare. Consequently, when this attenuated risk represents a harm, all other things being equal, they will be less risk-averse than they would otherwise be. This behavior may also contribute to phenomena such as postsurgery opioid overprescription (Thiels et al., 2017). Underweighting is most pronounced with very limited experience because a rare event is particularly unlikely to arise in a small sample. But as-if underweighting of rare events can occur even with ample experience—for instance, when a physician’s small sample of recent relevant experience has more sway than their ample past experience (e.g., because it is better remembered) or when a physician has ample experience but relies on just a few episodes (see Stewart et al., 2006) when making a decision. Finally, a cognitive process according to which a person makes choices by focusing on similar clusters of experience also implies reliance on small samples (Plonsky et al., 2015).

#### As-if overweighting of rare events: the hot-stove effect and experiential refractory periods

Although, on the aggregate level, as-if underweighting of rare events in experience is a robust pattern (see Hertwig et al., 2019), experiential dynamics can also create the opposite effect. The *hot-stove effect* refers to a behavior that initially yielded an extremely adverse outcome and can therefore give rise to a powerful behavioral bias that prevents the organism from repeating this behavior (Denrell, 2005, 2007; Denrell & March, 2001). A cat that sits on a hot stove lid may never approach another one, regardless of its temperature (Twain, 1897, p. 124). The cat behaves as if it overweights the (possibly) rare event of getting burned. The likelihood of a hot-stove effect depends on a number of factors, such as whether the organism can take precautionary actions (e.g., turning off the stove), the causal model the organism uses (e.g., believing that lightning never strikes twice), and the kind of feedback they receive (e.g., whether they get feedback on what would have happened had they chosen the forgone action). On a related note, the Depression-babies effect (Malmendier & Nagel, 2011) describes the phenomenon that people who live through macroeconomic shocks (e.g., the Great Depression) subsequently take fewer financial risks. Experimental research has also demonstrated that extreme negative outcomes, once experienced, may have disproportionate sway on people’s decisions (Lejarraga, Woike, & Hertwig, 2016; Ludvig et al., 2014; Spitzer et al., 2017), making people more risk-averse then they would otherwise be. The coronavirus pandemic may be such a negative outcome that profoundly will change the risk preferences of those who live through it, especially if the pandemic directly affected their health or financial well-being.

It is noteworthy that experience-induced risk aversion for harmful events can be transient, especially if the action in question is unavoidable (Le Mens & Denrell, 2011) or a person has the chance to observe outcomes for an option they did not choose. After an action has resulted in harm, a person is likely to be on the alert and behave as if they overweight the risk, at least during what could be called an “experiential refractory period” (for the notion of a wavy recency effect, see also Plonsky et al., 2015). The duration of this period likely depends on factors such as the magnitude of the experienced harm. For instance, a study of young drivers found that the likelihood that a driver would engage in behaviors indicative of risky driving (i.e., rapid starts, hard stops, and sharp turns) dropped significantly after a severe collision compared with a precollision period, that this difference persisted for at least 2 months after this event (O’Brien et al., 2017), and that psychological distress remained elevated for up to 3 years after the collision if they were injured (Craig et al., 2016). In the third month after the collision, risky behaviors rebounded significantly (although remaining lower than before the collision), although both groups of drivers—those with and without experience of a collision—displayed a gradual decrease in the rate of risky behavior across time. One interpretation of the rebound effect is that the psychological impact of the rare event appears to wane as the driver accumulates safe driving experience after the collision.^2^ Of course, there are likely to be substantial individual differences in how people respond to accidents (Spektor & Wulff, 2021).

### Experience and description: Does one overrule the other?

The third epistemic state (Fig. 4, top right) features both description and experience. This could be the epistemic state of a physician who has read the MMR health statistics and has a wealth of experience administering the vaccine, or of a person who is aware of statistics on the risk of sexually transmitted diseases and has had unprotected sex. Do these two types of risk representation—description and personal experience—integrate, or does one drown out the other? It is commonly thought that descriptive warnings are often ignored and may even backfire (Andrews, 2011; Steinhart et al., 2013). Meta-analyses on the efficacy of warnings have highlighted factors that shape their success, such as intended behavioral outcome, audience characteristics, message content, and delivery modes (e.g., Argo & Main, 2004; Purmehdi et al., 2017). Although such analyses are important for designing more effective warnings, it is also important to consider the target audience’s experiential starting point.

In the context of syphilis, for example, the proper use of condoms reduces the risk of contracting the disease. But a warning about the risks of unprotected sex may run counter to a person’s experience of having had unprotected sex without negative repercussions. Indeed, in 2018, there were 10.8 cases of syphilis per 100,000 people in the United States (Centers for Disease Control and Prevention, 2019a). Assuming that one needs to come into direct contact with a syphilis sore just once to become infected, one would need to have sex with 6,418 people to reach a 50% probability of contracting syphilis. Experience of safe encounters can thus potentially thwart a warning’s ability to shape behavior. This dynamic may also help explain why early climate-change warnings were relatively ineffective (Weber, 2006; Weber & Stern, 2011).

Several key factors determine the relative impact of description and experience in decision-making. Timing is one: When a warning coincides with the start of a decision-making process, it receives more weight than when it follows safe experiences (Barron et al., 2008). Warnings at the outset of a decision-making process can also induce safer behaviors in future decisions because the first instance is established as the default. Complexity is another factor: The impact of description on experience-based choice decreases when the tasks—and thus the task descriptions—become too complex (Weiss-Cohen et al., 2018; see also Lejarraga, 2010). Generally speaking, experience often seems to take precedence over description, which sometimes gets ignored altogether in decision-making (Erev et al., 2017; Lejarraga, 2010; Lejarraga & Gonzalez, 2011; Weiss-Cohen et al., 2016). In a powerful analysis of 14 choice anomalies (e.g., reflection effect, certainty effect, Petersburg paradox), most of the well-known description-based choice phenomena were found to be eliminated or reversed after a few experienced-based choices with feedback; the authors concluded that “the quantitative effect of experience can be large . . . even when the decision makers can rely on complete description of the incentive structure” (Erev et al., 2017, p. 393; see also Jessup et al., 2008; Lejarraga & Gonzalez, 2011). Finally, moving from the lab to the field, a review of the risk perception of natural hazards showed that personal experience and the lack thereof constitute “a strong factor in risk perception” (Wachinger et al., 2013, p. 1059).

### Neither experience nor description: unknown territory

In this epistemic state, neither descriptions nor experience exist (Fig. 4, bottom left). This state is perhaps best captured by the notion of “unmeasurable uncertainty” initially developed by Knight (1921) and Keynes (1937, 1973; see also Kozyreva & Hertwig, 2021). Unmeasurable uncertainty arises when there is no valid system of classification and no empirical evidence on the basis of which numerical measures can be assigned to one’s degrees of belief. This is unknown territory—no experience has been gathered and no description of the probability structure of the risky phenomenon in question is possible. Here, research on the description–experience gap is mute. This may have been the epistemic state in which researchers in Wuhan found themselves when reports of a new infectious disease (COVID-19) began to emerge. Initially, there was no valid basis for testing and classifying patients or tabulated evidence allowing epidemiologists to judge the disease’s key parameters. In situations of unmeasurable uncertainty, one may hope to draw on simple heuristics (e.g., win-stay, lose-shift) and on knowledge gathered in the past or by others (vicarious learning). But any kind of mapping and similarity relationship—is the new virus more like a common cold or more like MERS?—or other cognitive process (e.g., analogical reasoning, construction of mental models) involves navigating the twilight of uncertainty.

## New Research Questions and Themes

Our key point is that human responses to risks—complex, sometimes contradictory, sometimes self-defeating—will be better understood and predicted if researchers begin to systematically examine and model the two modes of learning about risk and their interplay. The description–experience framework we have outlined suggests a number of interesting lines of research, which we discuss in the following sections.

### *Cherchez l’expérience*: a heuristic for understanding perplexing risk behaviors

Examples of puzzling human responses to risk abound. South Koreans who have vigilantly fought COVID-19 while blithely brushing aside the prospect of nuclear annihilation are just one example (Sang-Hun, 2020). And it is often the case that experts and laypeople do not see eye to eye about a given risk (e.g., Bostrom, 1997; Sjöberg, 1999; Slovic et al., 1985). For instance, nearly 800,000 residents live in the red zone of Mount Vesuvius, Europe’s “ticking time bomb” (Barnes, 2011, p. 140), ignoring both dire warnings from volcanologists (Mastrolorenzo et al., 2006, 2010) and the incentive of cash payouts for moving (Barberi et al., 2008; Bruni, 2003).

One approach to understanding these calm responses in the face of possible Armageddon is to analyze individual and collective experience with the risk in question. For instance, most residents in the red zone have never experienced Mount Vesuvius erupting—the last eruption was in March 1944. One illustrative but admittedly simplistic way to think about the impact of such a long “all-clear experience” on a person’s risk beliefs is in terms of Laplace’s rule of succession.^3^ For instance, “modeling” the experience of a resident who has lived in the red zone for the past 20 years, the rule would suggest that the resident can be nearly certain (.999) of the next day being another “all-clear” day.

Given people’s seemingly perplexing behaviors in the face of risk, including the striking divergences between experts and laypeople, a promising research heuristic would be to consider the parties’ history of experience. At least three structural properties of experience may guide and inform such a “historical” analysis.

#### Asymmetry in experience

Both experts and laypeople can have—and are likely to have—different states of personal experience of a risk (and its nonoccurrence): from no experience at all to ample experience. For instance, a physician may regularly administer a medical intervention (e.g., a vaccine) and accumulate experience with its harms and benefits, whereas a patient may not have had a single encounter with the intervention. Yet this asymmetry may also be reversed—for example, when a physician prescribes a medication for a rare disease for the first time but the patient has many years of daily experience with the medication and its benefits and harms. Such experiential asymmetries are likely to be at the root of many expert–laypeople disagreements. They also imply that experiential expertise does not necessarily coincide with expert status.

#### A risk’s statistical nature and the accumulation of experience

**Fig. 6.**
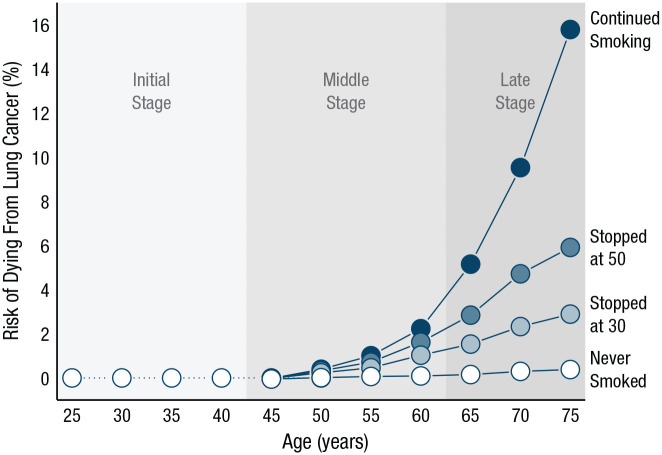
Stages of experience. The cumulative risk of dying from lung cancer is graphed as a function of age (up to age 75), separately for those who never smoked and those who quit at various ages. Data are from Peto et al. (2000) and represent death rates for men in the United Kingdom in 1990. Initial, middle, and late stages indicate the periods during which the risk of dying of lung cancer is absent, emerging, and in full effect, respectively.

Insight into the statistical nature of the risk in question can be very helpful when it comes to describing a person’s experiential history and even predicting their experiential future: How frequent or rare is the risk? Is its growth linear or exponential? How severe are its consequences? What is the delay between exposure to the risk and the experience of consequences? Is there cumulative risk with repeated exposure? For instance, smoking carries cumulative risks (see, e.g., Bosetti et al., 2008). Figure 6, which is based on data from Peto et al. (2000), plots the percentage cumulative risk of dying from lung cancer associated with smoking for men of different ages and depending on when and if they quit. In the initial stage of experience, the large majority of smokers will share the same experience: There is no great cause for concern. This uniformity of experience changes with increasing age (i.e., length of exposure): The risk of dying from lung cancer increases much more steeply with age for those who previously smoked than for nonsmokers, even more so for those who continue smoking into old age. Slovic (2000a, 2000b; see also Slovic et al., 1978) suggested that the initial window of an all-clear experience may explain why young smokers perceive themselves as being at little to no risk from each cigarette smoked, especially if they have not yet experienced the difficulty of quitting.

#### The dynamic nature of experience

**Fig. 7.**
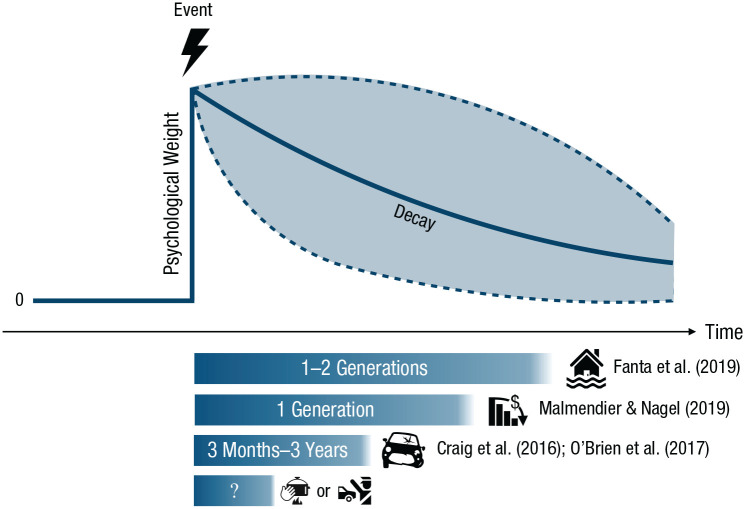
The prototypical time course of psychological weight assigned to an event by an individual or a group in response to having experienced it. Psychological weight decays differently depending on the type of event. For instance, decay in collective memory of cultural goods, such as music, typically follows a biexponential form (Candia et al., 2019), whereas individual memory decay is typically assumed to follow a simple exponential form (Schooler & Hertwig, 2005). Empirical evidence also highlights that memory biases in favor of extreme events contribute to the description–experience gap (Madan et al., 2017).

Experience and its potential sway on behavior is not static. Traumatic experiences of a catastrophic event—a crime, a financial disaster, a life-threatening disease—can have lasting impact. Even after their repercussions have receded, these experiences shape behavior (remember the Depression-babies effect; Malmendier & Nagel, 2011), especially when new experiences that might offset the traumatic experience are systematically avoided (Denrell, 2005, 2007; Denrell & March, 2001) or otherwise unavailable. One important research avenue for the future is therefore to track the temporally dynamic psychological weight of a risk event across time (Fig. 7). For instance, Fanta et al. (2019) examined 1,300 settlements and their experience of major floods and found that “respect for floods waned in the second generation” (p. 2), which is when people moved from safer sites back toward to the river.^4^

### Experiential traps: When and why do descriptive risk warnings fail?

Another insight offered by the description–experience framework is that warnings need to work for people with very different degrees of experience—from none at all to ample experience with the risk in question. This probably explains why one-size-fits-all warnings fail for at least some portion of the audience. As Barron et al. (2008) demonstrated experimentally, warning effectiveness can be reduced when recipients have previously had safe (and possibly highly enjoyable) experience with a (rare) risk before being warned—for instance, having had unprotected sex without experiencing negative health consequences or having illegally downloaded copyrighted material without being caught. Early warnings (i.e., before the accumulation of “safe” experiences) may have the desired effect; however, their effects may wane if they are followed by many safe experiences. Zohar and Erev (2007) proposed this tendency to be a key reason why workplace accidents and injuries continue to occur despite ample warnings, regulations, and personal protective equipment; in 2019, 2.8 million nonfatal workplace injuries and illnesses occurred in the private industry in the United States, and one worker died every 99 min from a work-related injury (Bureau of Labor Statistics, 2020). The warning of a risk (e.g., hazardous noise levels at work) and its potentially delayed consequences (e.g., hearing loss, tinnitus) inevitably competes with repeated experience of seemingly safe exposure to that risk. The more that experience accumulates, the more likely it is to eclipse the effects of the warning and to undercut compliance with safety measures—even more so when disregarding the warning allows workers to get their routine tasks done faster and more comfortably.

These considerations raise a number of questions for future research. For example, what is the optimal timing of a warning? And how can warnings be adapted to flexibly fit the experiential state of the audience? There appears to be a basic regularity that (a) the less experience people have with a task, the stronger a description’s impact on behavior, and (b) more experience—especially of safe episodes—attenuates the effect of warnings (Barron et al., 2008; Weiss-Cohen et al., 2021^5^). This dynamic may change if warnings explicitly acknowledge people’s state of experience and if they introduce and explain the “ambiguities of experience” (March, 2010, pp. 106–109) and “experiential traps” such as the lure of experience of safe episodes (before and after a warning) and time delays in the manifestation of a risk.

Another potential trap is that the rarity of a risk often implies a “primacy halo” effect: Even if the probability of a risk occurring in a given time period is very small (e.g., a fatal car accident on a single trip), the probability of at least one risk event actualizing becomes much larger over repeated exposure to that risk (e.g., a lifetime of car trips; Slovic et al., 1978). This means again that, on average, multiple safe episodes especially at the outset of a sequence of experiences carry the danger of a risk being underestimated.

In our view, it is important to acknowledge and study the real-world consequences of such experiential traps. A better understanding of these patterns may help risk researchers and public-health officials to anticipate behaviors that are otherwise difficult to fathom, highly undesirable, and possibly preventable. Take, for instance, another experiential trap that may be dubbed the “curse of success”: the erosion of public support for preventive health measures that prove successful. This phenomenon has been observed in the context of the coronavirus pandemic (Cayetano & Crandall, 2020) and more generally in declining rates of vaccination uptake (United Nations Children’s Fund, 2019). In the first 6 months of 2019, 364,808 measles cases were recorded across 182 countries—the highest figures since 2006 (Korn et al., 2020). According to the World Health Organization (2019), vaccine hesitancy was one of the ten major threats to public health in 2019. Vaccine hesitancy may be due, at least in part, to an unfortunate collusion of experience and vaccine-critical descriptions. Thanks to the success of preventive measures taken in previous decades, few people in recent generations have experienced measles or its effects. From this experience, they may conclude either that vaccination is a successful public-health strategy that should be continued or that measles is nothing to worry about. In addition, the description–experience framework would suggest that people may overweight the small risk of serious side effects of vaccination, which further erodes their willingness to vaccinate.

### When is description discounted?

As much as experience appears key to how people respond to risks, descriptions and the psychological processes underlying their effects also matter. Numerous factors that weaken the impact of description-based risk communication have been identified, including repeated exposure (e.g., to alcohol warning labels or graphic visual tobacco warnings; Kim & Wogalter, 2009) or nontransparent presentations of health statistics (Gigerenzer et al., 2007). Warnings that could be perceived to threaten personal freedoms may also backfire because they induce stress and reactance (Hall et al., 2016). Such perceptions can trigger the urge to reclaim one’s freedom by engaging in the potentially harmful behavior (Pham et al., 2016).

In other cases, warnings may inadvertently invoke a tangible experiential dimension, the behavior of people around us. Many warnings begin by identifying the undesirable behavior and noting its high prevalence. For instance, “61% of Americans have no money saved for their healthcare expenses” (Business Wire, 2018, para. 1), or “most Americans with diabetes skip annual sight-saving exams” (Knutsen, 2019, para. 1). By using such framings, warnings communicate descriptive social norms—perceptions of what most other people actually do—and may thus send the message that “if a lot of people are doing this, it’s probably a wise thing to do” (Cialdini, 2007, p. 264). Thus, by describing the frequency of the very behavior it aims to change, a warning may normalize that behavior and thus prove counterproductive (Schultz & Tabanico, 2009).

### Can simulated experience enrich risk communication?

As we have shown, personal and direct experience of a risk can be misleading, especially (but not only) if the risks are rare, delayed, and noisy and there is no feedback on risk events that did not occur. But experience may also offer a solution in situations in which descriptions fail. In a letter to his siblings, the English poet John Keats wrote, “Nothing ever becomes real till it is experienced—Even a Proverb is no proverb to you till your Life hast illustrated it” (1819/2000). If it were strictly true that only experience can lend realism to words, thoughts, and descriptions, this would bode ill for any attempt to communicate the danger of risks through descriptions—especially to audiences who have not yet experienced them or when the risk to the individual remains opaque and unobservable (e.g., the risk of overdiagnosis; see Carter et al., 2015). However, research showing that warnings can successfully communicate benefits and risks if they are appropriately designed on numerous dimensions (Andrews, 2011) suggests that Keats’s words should not be taken to mean that descriptions of risk are doomed to fail.

**Fig. 8.**
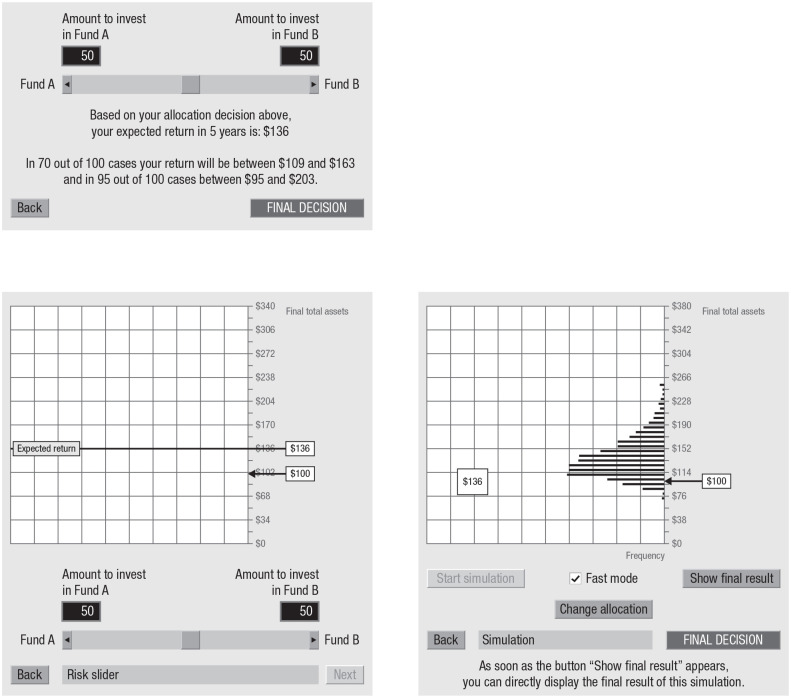
The description and risk-tool conditions, as examined by Kaufmann et al. (2013). Participants were asked to allocate an endowment of $100 between two funds: the risk-free fund A and the risky fund B. In the description condition, participants were given a numeric description stating the expected return. Participants read the description after making an allocation (in percentages) with the risk slider and could try different allocations (and read about their effects) before deciding on a final one. In the risk-tool condition, participants made an allocation with the risk slider and saw the simulated expected returns of their investment on a graphical interface. To simulate experience sampling, participants used the tool to draw potential returns randomly from a distribution based on historical data. Each random draw contributed to a sampling distribution function that was displayed gradually on the screen. Adapted with permission from “The Role of Experience Sampling and Graphical Displays on One’s Investment Risk Appetite,” by C. Kaufmann, M. Weber, and E. Haisley, 2013, *Management Science*, Volume 59, pp. 337–338.

Sometimes, however, experience can make things feel more real or foster better insight than description—and it may be worth harnessing this quality in risk communication. Indeed, inspired by work on the description–experience gap, researchers have recently begun to investigate the potential benefits of “simulated experience” (e.g., Armstrong & Spaniol, 2017; Hogarth & Soyer, 2015; Wegier et al., 2019; Wegier & Shaffer, 2017) for communicating statistical information about risks. For example, financial institutions that offer investment products are by law required to provide information about the key properties of those products (e.g., risks, costs, past performance history). This information can be presented to clients in different ways. The default approach is in terms of numerical and graphic descriptions (e.g., historical returns in fact sheets). A very different approach is for clients to interactively sample possible outcomes, that is, possible returns on an investment—what Kaufmann et al. (2013) called “experience sampling.” Each sampled outcome contributes to the return distribution, which is displayed at the end of the sampling process. Kaufmann et al. (2013) designed a simple “risk tool” that enabled undergraduate students at a German university to experience the distribution of a risky financial product. Figure 8 represents the description condition and the experienced-sampling condition that they examined (along with two other conditions that are not addressed here). The authors observed differences between experience and description on various dimensions. For instance, experienced-sampling investors allocated a larger percentage of their endowment to the risky fund than did description investors; they were more accurate in their understanding of the options’ expected return and, importantly, in the estimated probability of a loss. Others have built on the work of Kaufmann et al. (e.g., Bradbury et al., 2015, 2019) and pointed to the boundaries of this intervention. Abel et al. (2021) recently found that a simple interactive game that experientially simulates the odds of winning the South African National Lottery via dice rolling (equivalent to rolling a six with all nine dice) appears to offer a way of giving people “brief experiences that correct biases in their beliefs” (p. 1).

The possibility of “simulated experience” raises a number of questions that are highly relevant for risk communication and for boosting people’s ability to navigate real-world risks (Hertwig & Grüne-Yanoff, 2017). For instance, which dimensions of the statistical properties of a risk are better communicated—meaning that the target audience gains more insight—through simulated experience than through description alone? Candidate properties here include information about the variance (volatility) of outcomes (as in Kaufmann et al., 2013), the rarity of events, the temporal dynamics of a process (e.g., the accumulation of risks with repeated exposure; Wegwarth et al., 2021), or exponential- versus linear-growth processes. In addition, which “qualia” of a risk—meaning the way things seem to decision makers (e.g., the perceived sensation of pain caused by a headache)—may be communicated and approximated through simulated experience but are lacking in descriptions? For instance, can the earthquake simulators used in disaster training centers throughout Japan and recommended for use elsewhere (e.g., in Nepal; Uprety & Poudel, 2012) convey the visceral dimensions of the threat and its swift temporal dynamic in a way that even the best descriptions cannot? Last but not least, simulated-experience scenarios may give people a more “realistic” sense of what they will encounter once a hazard strikes, as well as space to practice the appropriate behaviors.

## Conclusions

The COVID-19 pandemic is a forceful reminder that coping with risks requires not only evidence-based protective interventions but also an informed and cooperative public that accepts and adheres to those interventions. Yet individual and public responses to risks are often perplexing and even maladaptive. One reason is that people’s mental models of risks are richer than those assumed in the common technological definitions (see Slovic, 1987). Another reason is that people’s knowledge of risks stems from two imperfect teachers: descriptions and experience. Each implies distinct ambiguities and psychological effects. We believe that a better understanding of the two, as well as of the effects that emerge when description and experience co-occur, will enrich the understanding of people’s responses to risks—as well as the ability to predict and guide those responses.

## Transparency

*Action Editor:* Joshua Hicks

*Editor:* Laura A. King

## Notes

1. As of March 17, 2021, South Korea had registered 96,849 coronavirus cases and 1,686 deaths relative to, for instance, Germany’s 2,594,675 cases and 74,431 deaths; see https://www.worldometers.info/coronavirus.

2. For an early and thorough discussion of the methodological problems of studies that examine the effects of personal experience on self-protective behavior but cannot randomly assign participants to accident or victim conditions, see Weinstein (1989).

3. Laplace’s rule of succession (see Zabell, 1989) states, in brief, that if an event (e.g., the sun’s daily rising) has occurred *m* times in successive trials, then the probability that this event will occur in the next trial is (*m* + 1)/(*m* + 2). Note that our use of Laplace’s rule as one way of enumerative induction is simply to illustrate the power of repetitive experience. We do not suggest that the rule’s assumptions are met in the lives of Neapolitans, namely, that their trials (days) are independent, that the occurrence or nonoccurrence of the event is equally likely to occur, or that their experience corresponds to sampling balls from an urn. Last, the rule does not take into account that residents are also exposed to expert warnings.

4. “Dynamic” can pertain to more than the systematically varying impact of the remembered experience with the passage of time. In behavioral-decision research (Edwards, 1962), dynamic decisions refer to decisions that involve sequences in which previous actions and their outcomes influence future states of the world and future actions. This dynamic quality of important decisions has not (yet) received much attention in the extant research on the description–experience gap, in which stationary payoff distributions are often studied.

5. In an experimental gambling paradigm in which people also received feedback about forgone payoffs, this relationship was reversed (Weiss-Cohen et al., 2021); however, information about forgone payoffs is rarely available outside controlled experimental settings.
